# Addressing Inpatient Hyponatremia Through Targeted Automatic E-consults: A Pilot Randomized Trial

**DOI:** 10.1007/s11606-024-09054-5

**Published:** 2024-10-22

**Authors:** Timothy J. Judson, Lowell Lo, John E. Demko, Michelle Mourad, Kathleen D. Liu, Robert M. Wachter, Isaac Lopez, Sri Lekha Tummalapalli

**Affiliations:** 1 Department of Medicine, University of California San Francisco, San Francisco, CA USA; 2 Maine Medical Partners Adult Hospital Medicine, Maine Medical Center, Portland, ME USA; 3 Division of Healthcare Delivery Science & Innovation, Department of Population Health Sciences, Weill Cornell Medicine, New York, NY USA; 4 Division of Nephrology & Hypertension, Department of Medicine, Weill Cornell Medicine, New York, NY USA

**Keywords:** e-consults, hyponatremia, inpatient, pilot trial, feasibility, acceptability, specialist consultation, targeted automatic e-consultation

## Abstract

**Background:**

Hyponatremia is the most common electrolyte abnormality in hospitalized patients. Treatment of hyponatremia is associated with improved outcomes, but more than one in three cases of new onset hyponatremia is not corrected by the time of hospital discharge. Nephrologist input may improve the diagnosis and treatment of hyponatremia, but specialist resources are limited. Targeted automatic electronic consultations (TACos) may be one approach to provide expert nephrologist guidance to the workup and management of hyponatremia using a scalable model.

**Objective:**

Evaluate the feasibility and acceptability of a TACo intervention for hospitalized patients with hyponatremia.

**Design:**

Single-site, parallel-group cluster randomized trial.

**Participants:**

Adult inpatients with hyponatremia on the hospital medicine service.

**Interventions:**

A nephrologist conducted TACos on intervention patients, making diagnostic and therapeutic recommendations daily (if warranted) until discharge or resolution of hyponatremia.

**Main Measures:**

Measures of feasibility included the number of eligible participants, percentage receiving TACos, number of TACos per participant, and percentage of formal nephrology consults. Acceptability was assessed by a post-intervention survey. Clinical outcomes, including the percentage of hyponatremia cases that resolved by discharge, were also assessed.

**Key Results:**

We identified 62 patients who met inclusion criteria: 38 in the intervention group and 24 in the control group. A nephrologist determined that 26 of 38 intervention patients (68%) would likely benefit from diagnostic and management recommendations; 67 TACos were performed (mean 2.6 per patient). Fourteen of 18 primary team physicians (78%) reported that the e-consults changed their management, and 15 of 18 (83%) wanted TACOs to continue. Resolution of hyponatremia, length of stay, 30-day readmissions, and costs were similar in the intervention and control groups.

**Conclusions:**

Inpatient TACos for hyponatremia were feasible and acceptable to primary teams, and frequently led to changes in diagnosis and management. Further studies are needed to determine the impact of the TACo model on clinical outcomes and cost-effectiveness.

**Supplementary Information:**

The online version contains supplementary material available at 10.1007/s11606-024-09054-5.

## INTRODUCTION

Hyponatremia is the most common electrolyte abnormality in hospitalized patients, with a prevalence of 15–30%.^1,2^ Acute and severe hyponatremia can have dire consequences and must be treated promptly.^3–5^ Moderate hyponatremia (125–129 mEq/L) is also associated with adverse outcomes and guidelines recommend treatment according to the chronicity and presence of symptoms.^4,6^ Studies across various patient populations have demonstrated improved outcomes after correction of hyponatremia, suggesting that inpatient clinicians should try to correct low sodium values when possible.^6–10^ Specifically, improvements in plasma sodium are associated with improved gait parameters and reaction times as well as decreased length of stay, 30-day readmissions, hospital costs, and mortality.^4,7–11^ Despite this evidence, more than one in three cases of acute-onset hyponatremia is not corrected by the time of hospital discharge.^4,8^ One approach to more consistently correcting hyponatremia in hospitalized patients is to provide expert nephrologist guidance into hyponatremia workup and management. However, specialist resources are limited and such consultations are not routinely obtained.

Targeted automatic e-consultation (TACo) is a model of proactive e-consultation based on automated identification of high-risk patients from the electronic health record (EHR).^12,13^ TACos are triggered by patients’ EHR data rather than a consultation request. Once a patient is identified, the consultant is presented with a customized EHR view of pertinent information, and can then choose to provide targeted advice, suggest formal consultation, or neither. The TACo model has been shown to improve clinical outcomes in glucose management, and has been proposed for several other conditions.^12–15^

Hyponatremia may be well suited for management through the TACo model, as hyponatremia is common, easily identified via objective criteria in the EHR, often managed suboptimally by primary teams, and may benefit from specialist recommendations for further testing or treatment. However, there have also been concerns raised about TACos, including the feasibility from a workflow and resources standpoint, the possibility that receiving unsolicited management advice may not be acceptable to primary teams, and the potential erosion of clinical expertise if generalists consistently receive specialist input when managing common problems.

The feasibility, acceptability, and clinical effectiveness of targeted, automated e-consults for inpatients with hyponatremia have not been described in the literature. In this pilot randomized trial, we sought to evaluate the feasibility and acceptability of TACos for hospitalized patients with hyponatremia. We also assessed the resolution of hyponatremia by discharge among patients receiving TACos and those receiving usual care.

## METHODS

### Study Setting

We conducted this study at University of California San Francisco (UCSF) Health, a large academic health system consisting of three campuses, with nearly 1000 inpatient beds and approximately 45,000 hospital admissions annually. UCSF uses a commercially available EHR from Epic Systems (Verona, WI). TACos have been performed for glucose management at UCSF since 2012.^14,15^ Traditional inpatient e-consults were introduced to select consult services in 2015.

### Study Design and Oversight

This study was a single-site, parallel-group cluster randomized trial. Inpatient primary medicine teams were randomized to intervention or control in a 1:1 ratio. Among 16 inpatient medicine teams, 8 were initially assigned to the intervention group and 8 to the control group. Teams that were co-located in the same work rooms were then re-assigned to the same group to minimize contamination (which occurred for 1 team). Patients and study investigators conducting chart reviews to ascertain outcomes were blinded to the treatment assignment. The study was conducted over a 6-month period from November 2020 to April 2021. Prior to initiating the study, the investigators informed the Division of Hospital Medicine and Internal Medicine residents by email about the design, rationale, and goals. The study was reviewed and approved by the UCSF Office of Human Research Protection. Informed consent was waived given minimal risk to participants.

### Study Population

Patients were included in the study if they met the following criteria: age ≥ 18, hospitalized on the medicine service in the acute care or transitional care units at UCSF’s Parnassus campus, and having 2 or more consecutive sodium values < 130 mEq/L during admission. Patients were excluded if they were on hemodialysis, already had an active nephrology consult, were receiving comfort care, or if the sodium was at baseline based on historical sodium trends and previous hyponatremia workups. Patients were considered to be at their baseline low sodium level if they had multiple sodium values (≥ 2) in the hyponatremic range during the past year that were not explained by acute, reversible episodes (e.g., offending medication that was then discontinued).

### Intervention Description

**Figure 1:**
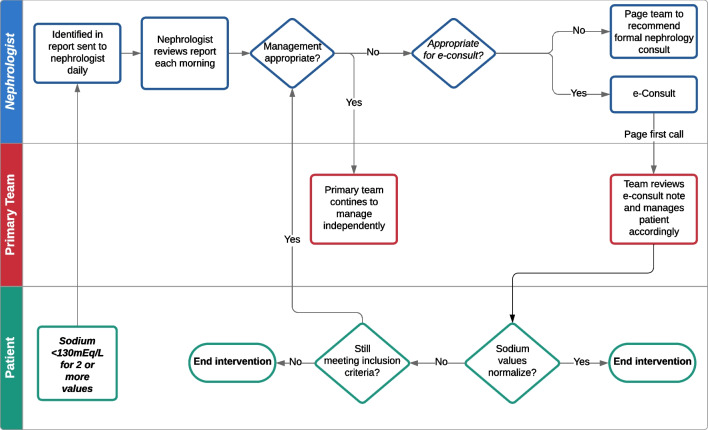
Intervention flowchart. Swimlane diagram showing the components of the hyponatremia-targeted automatic E-consult intervention. Rectangles represent steps in the process; diamonds represent decision points; ovals represent endpoints.

**Figure 2:**
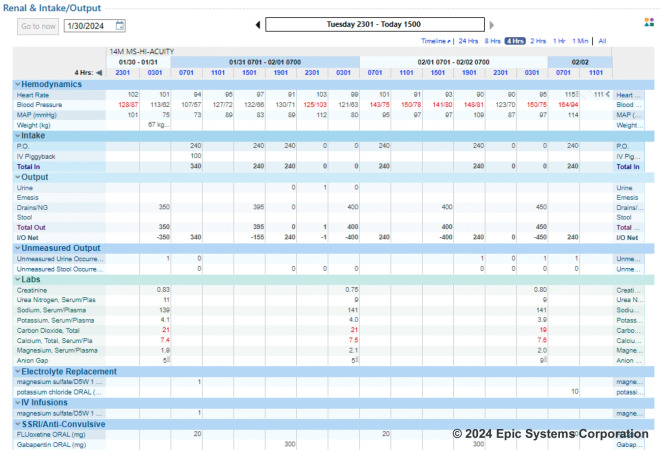
Screenshot of hyponatremia EHR dashboard. Example of the custom dashboard used by the study nephrologist to conduct hyponatremia E-consults.

**Figure 3:**
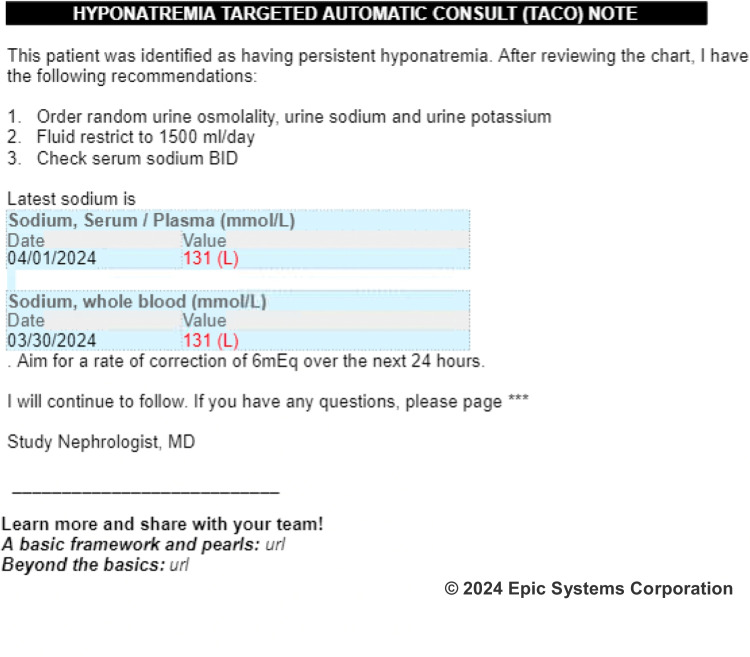
Screenshot of sample-templated e-consult note for hyponatremia intervention.

Figure 1 summarizes the intervention design. Each morning, the study nephrologist (LL) received an automatically generated report from the EHR listing patients who met inclusion criteria in the previous 24 h. After confirming that the patient met none of the exclusion criteria by reviewing a structured spreadsheet and the patient’s chart, the nephrologist was informed whether the patient was randomized to the intervention or control groups (based on their primary team). The control group received usual care for hyponatremia, with management by the primary team and consultation of specialty services (including nephrology) at the team’s discretion. For participants in the intervention group, the study nephrologist conducted a targeted chart review using a custom-built EHR dashboard to determine whether the patient’s care could be enhanced through the provision of diagnosis and/or management recommendations where indicated, and whether recommendations could be provided by e-consultation or required formal consultation. The dashboard included intake and output data, weights, medications, vital signs, and select lab values and trends (Fig. 2). If the study nephrologist had no recommendations (e.g., because the primary team had already ordered all necessary diagnostic tests and was managing hyponatremia appropriately), that was indicated in the study documentation but the nephrologist left no note in the patient’s chart. If the nephrologist had recommendations, they wrote a templated TACo note and paged the “first-call” physician to inform them of the e-consult and recommendations (Fig. 3). In addition to management recommendations, initial notes often included recommendations for the team to collect additional data (e.g., daily weights, urine studies). Teams sometimes called back for clarification, but a phone call was not required. If the study nephrologist determined the patient in the intervention group required a formal nephrology consult, they communicated this recommendation to the primary team via a page. If the nephrologist left e-consult recommendations for a patient, they added the patient to a list, followed them daily and left recommendations as needed until either (1) sodium increased to ≥ 135 mEq/L or returned to baseline, (2) the patient was discharged, or (3) the patient no longer satisfied inclusion/exclusion criteria (e.g., due to transfer to the intensive care unit). All TACo notes included educational materials including links to (1) a one-page framework for hyponatremia diagnosis and management (supplemental Fig. 1), and (2) a six-part online course on hyponatremia available for free to any UCSF employee.

### Participant Characteristics

Baseline characteristics were gathered from the Epic Clarity database and included age, sex, race, ethnicity, primary language, insurance type, home address, and admission diagnosis. Patient addresses were geocoded to census block groups using the US Census Bureau website and linked to California state-level Area Deprivation Indices from 2021.^16–18^ Admission diagnoses were manually classified into ICD-10 diagnosis code categories.

### Feasibility and Acceptability

Measures of feasibility included (1) the number of participants eligible for inclusion in our study (screening and recruitment), (2) the percentage of eligible participants receiving TACo recommendations (recruitment), (3) the number of TACos per participant (intervention fidelity), and (4) the percentage of participants receiving a formal nephrology consult (balance measure).^19^ To assess acceptability, a survey (supplemental Fig. 2) was emailed to each of the “first-call” physicians for both intervention and control participants via an anonymous Qualtrics link. For physicians whose patient received a hyponatremia TACo, the survey assessed satisfaction with the e-consult and knowledge of hyponatremia management. For physicians whose patient was assigned to the control group, the survey asked whether they would like to receive TACos for hyponatremia, and assessed knowledge of hyponatremia management using the same questions posed to the intervention group physicians. A single email reminder was sent 3 days after the initial survey was sent.

### Exploratory Outcomes

Additional outcomes assessed included (1) hyponatremia resolution at discharge (Na ≥ 135 mEq/L), (2) the lowest sodium value (mEq/L), (3) time to hyponatremia resolution in days, and (4) the rate of sodium correction (mEq/L/day). Outcomes related to the hospitalization included (1) length of stay (days), (2) number of 30-day readmissions, and (3) cost of hospital stay ($USD). A nephrologist not involved in the intervention (JD) performed blinded chart reviews of intervention and control participants > 30 days after enrollment to ascertain outcomes.

### Statistical Analysis

Patient characteristics for the intervention group, control group, and total cohort were reported as percentages for categorical variables and median [interquartile range] for continuous variables. Feasibility and acceptability measures were reported as percentages. Resolution of hyponatremia and hospitalization outcomes were compared in the intervention vs. control groups using chi-squared tests for categorical variables and Wilcoxon rank-sum tests for continuous variables. The primary analysis was intention-to-treat, and an as-treated analysis, which excluded intervention group participants who did not receive a TACo, is presented in the supplementary data. Data analysis was performed using Stata version 15.1 (StataCorp).

## RESULTS

### Patient Characteristics

**Figure 4:**
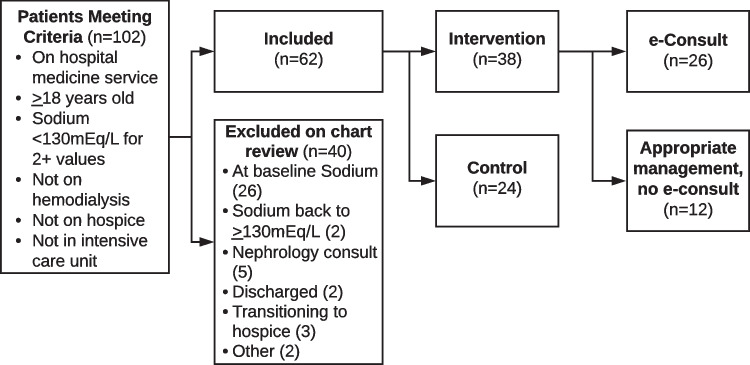
Study CONSORT (flow) diagram.

A total of eight primary medicine teams were randomized to the intervention group and eight to the control group. During the study period, 102 patients met EHR-based inclusion criteria and were screened for eligibility (Fig. 4). During chart review, 40 patients were excluded due to serum sodium levels that were low but were at baseline (*n* = 26), sodium levels already ≥ 130 mEq/L (*n* = 2), existing formal nephrology consult (*n* = 5), discharged at time of identification (*n* = 2), transitioning to hospice (*n* = 3), or other (*n* = 2). Of the remaining 62 patients, 38 were on intervention teams, while 24 were on control teams.

Patient characteristics between the intervention and control groups are shown in Table 1. Median age was 67 in the intervention group and 61 in the control group. Fifty-eight percent of control group patients were male compared to 46% in the intervention group. Race and ethnicity in the intervention vs. control groups was notable for 32% vs. 46% non-Hispanic White, and 42% vs. 38% Asian, respectively. Fewer patients in the intervention vs. control group had English as a primary language (66% vs. 88%) and commercial insurance (18% vs. 42%). A total of 83% of patients resided in census block groups in the first or second Area Deprivation Index decile in the state, indicating high neighborhood-level socioeconomic status. Patients were most often admitted for diseases of the digestive system (21%); endocrine, nutritional, and metabolic diseases (15%); diseases of the respiratory system (11%); and infections (10%).

**Table 1:** Patient Characteristics in the Intervention and Control Groups, Intention-to-Treat Analysis

	Intervention group (*n* = 38)	Control group (*n* = 24)	Total (*n* = 62)
Age	66.5 [61–77]	61 [57–68.5]	64.5 [59–75]
Sex			
Male	22 (58%)	11 (46%)	33 (53%)
Race and ethnicity			
Non-Hispanic White	12 (32%)	11 (46%)	23 (37%)
Non-Hispanic Black	2 (5%)	1 (4%)	3 (5%)
Hispanic or Latino	6 (16%)	2 (8%)	8 (13%)
Asian	16 (42%)	9 (38%)	25 (40%)
Native Hawaiian or Other Pacific Islander	1 (3%)	0 (0%)	1 (2%)
American Indian or Alaska Native	0 (0%)	0 (0%)	0 (0%)
Other, unknown, or declined	1 (3%)	1 (4%)	2 (3%)
Primary language			
English	25 (66%)	21 (88%)	46 (74%)
Non-English	13 (34%)	3 (13%)	16 (26%)
Insurance			
Commercial	7 (18%)	10 (42%)	17 (27%)
Medicare	26 (68%)	7 (29%)	33 (53%)
Medicaid	5 (13%)	7 (29%)	12 (19%)
Area Deprivation Index decile*	1 [1, 2]	2 [1–3]	2 [1, 2]
Admission diagnosis category**			
Certain infections and parasitic diseases	3 (8%)	3 (13%)	6 (10%)
Neoplasms	4 (11%)	1 (4%)	5 (8%)
Diseases of the blood/blood-forming organs and certain disorders involving the immune mechanism	1 (3%)	0 (0%)	1 (2%)
Endocrine, nutritional, and metabolic diseases	5 (13%)	4 (17%)	9 (15%)
Mental, behavioral, and neurodevelopmental disorders	1 (3%)	0 (0%)	1 (2%)
Diseases of the nervous system	1 (3%)	3 (13%)	4 (6%)
Diseases of the eye and adnexa	1 (3%)	0 (0%)	1 (2%)
Diseases of the circulatory system	2 (5%)	2 (8%)	4 (6%)
Diseases of the respiratory system	5 (13%)	2 (8%)	7 (11%)
Diseases of the digestive system	7 (18%)	6 (25%)	13 (21%)
Diseases of the musculoskeletal system and connective tissue	0 (0%)	3 (13%)	3 (5%)
Diseases of the genitourinary system	4 (11%)	0 (0%)	4 (6%)
Symptoms, signs, and abnormal clinical laboratory findings, not elsewhere classified	3 (8%)	0 (0%)	3 (5%)
External causes of morbidity	1 (3%)	0 (0%)	1 (2%)

Age and Area Deprivation Index decile presented as median [interquartile range]. Percentages may not add to 100% due to rounding

^*^State decile. Nine participants had missing Area Deprivation Indices (ADI). Decile 1 is the lowest ADI (least disadvantaged) and 10 is the highest ADI (most disadvantaged)

^**^Zero participants had admission diagnoses in the following categories: diseases of the ear and mastoid process; diseases of the skin and subcutaneous tissue; pregnancy, childbirth, and puerperium; certain conditions originating in the perinatal period; congenital malformations, deformations, and chromosomal abnormalities; injury, poisoning, and certain other consequences of external causes; factors influencing health status and contact with health services

### Feasibility and Acceptability

After initial chart review, the e-consulting nephrologist determined that 26 of 38 patients (68%) would benefit from specialist recommendations. The remaining 12 patients assigned to the intervention group did not receive e-consults because existing management was felt to be appropriate. The 26 patients who received hyponatremia TACos received a total of 67 hyponatremia e-consults (mean, 2.6 TACos per patient).

Surveys were sent to the providers for all 26 patients who received hyponatremia TACos. Of these, 18 responded to the survey (response rate of 69%). Of the respondents, 17 (94%) were aware that the TACo took place. Fourteen of 18 (78%) reported that the TACo changed their management by prompting them to order additional diagnostic tests (5 of 18; 24%) and/or changing their management plan (12 of 18; 57%). A total of 13 of 18 (72%) respondents reported that the TACo either greatly improved or slightly improved their understanding of the workup and management of hyponatremia, and 15 respondents (83%) definitely or probably wanted these TACos to continue.

A separate survey was sent to 24 providers of control patients who did not receive hyponatremia TACos. Of these, 17 responded (response rate of 70%). Of these, only one team (5.9%) reported formally consulting the nephrology service. Four respondents (23.5%) would have liked to have a nephrologist proactively leave recommendations.

Recipients of TACos answered 90% of hyponatremia knowledge questions correctly, while residents/attendings who did not receive e-consults answered 86% of questions correctly (*p* = 0.719).

### Hyponatremia Resolution and Hospitalization Outcomes

There were no significant differences in hyponatremia resolution or hospitalization outcomes between the intervention and control groups (Table 2). The proportion of patients in whom hyponatremia had resolved to ≥ 135 mEq/L by time of discharge was 42% in the intervention group and 29% in the control group (*p* = 0.304). There was also no statistically significant difference between groups in the sodium nadir during hospitalization (median 125 mEq/L vs. 126 mEq/L, *p* = 0.321), time to hyponatremia resolution (median 3.7 vs. 2.9 days, *p* = 0.707), and rate of correction (median 1.97 vs. 2.24 mEq/L/day, *p* = 0.989). Median length of stay was 7 days [IQR 4–15] in the intervention group and 6 days [IQR 3.5–14] in the control group. Number of 30-day readmissions per patient was an average of 0.50 [standard deviation 0.76] in the intervention group and 0.42 [standard deviation 0.58] in the control group. Median total cost of the hospitalization was $26,802 [IQR $15,840–65,308] for intervention patients and $25,222 [IQR $13,308–62,979] for control patients. When excluding patients who did not receive a hyponatremia e-consult because the study nephrologist had no additional recommendations (as-treated analysis), the results were similar (Supplemental Table 2).

**Table 2:** Hyponatremia Resolution and Hospitalization Outcomes, Intention-to-Treat Analysis

	Intervention group (*n* = 38)	Control group (*n* = 24)	*p*-value
Hyponatremia resolution			
Resolved at discharge ≥ 135 mEq/L (%)	16 (42%)	7 (29%)	0.304
Lowest sodium value (mEq/L)	125 [124–127]	126 [124–128]	0.321
Time to hyponatremia resolution ≥ 135 mEq/L (days)	3.7 [2.1–7.4]	2.9 [2.3–4.8]	0.707
Rate of correction to ≥ 135 (mEq/L/day)	1.97 [1.29–3.65]	2.24 [0.91–3.68]	0.989
Hospitalization outcomes			
Length of stay (days)	7 [4–15]	6 [3.5–14]	0.612
Number of 30-day readmissions/patient	0.5 [0.76]	0.42 [0.58]	0.885
Cost of hospital stay ($USD)	$26,802 [15,840–65,308]	$25,222 [13,308–62,979]	0.908

Continuous variables presented as median [interquartile range] except for readmissions (presented as mean [standard deviation]). Intervention and control groups were compared using chi-squared tests for categorical variables and Wilcoxon rank-sum tests for continuous variables

## DISCUSSION

This pilot randomized trial demonstrated the feasibility and acceptability of a structured, targeted, automatic nephrologist e-consultation (TACo) for hospitalized patients with hyponatremia. We found that, when patients with hyponatremia were triaged for nephrologist review, the specialist determined that greater than two-thirds of patients with hyponatremia would likely benefit from nephrology recommendations. When recommendations were provided, primary teams reported that they changed management in 78% of cases, and 83% of primary teams were proponents of these automatic e-consults continuing.

Although a greater percentage of study patients had resolution of their hyponatremia by the time of discharge, this difference was not statistically significant, nor were there any statistically significant differences in other outcomes including lowest sodium value, time to hyponatremia resolution, rate of correction, length of stay, 30-day readmissions, or cost of the hospitalization. Importantly, the study was not powered to detect modest changes in clinical effectiveness outcomes.

This study is the first, to our knowledge, to assess the feasibility, acceptability, and clinical effectiveness of automatic inpatient e-consults for hyponatremia. These findings suggest that the intervention is acceptable to primary teams and feasible to administer in a large, academic hospital setting. The finding that after chart review, the nephrologist made new recommendations for more than two-thirds of patients is consistent with previous findings suggesting undertreatment of hyponatremia in the hospital setting.^8,20^ These results are consistent with those described by Rushakoff et al., who determined that a TACo framework for inpatient glucose management was feasible and acceptable to primary teams, and led to educational gains.^14,15^

The clinical effectiveness of inpatient e-consults for hyponatremia is still unknown, as this study was not powered to detect differences in clinical outcomes, and we are unaware of any prior studies assessing inpatient hyponatremia e-consults. While it is recommended by expert panels that nephrologists consult on patients with severe hyponatremia,^4^ it is unknown whether their early involvement for patients with mild/moderate hyponatremia will improve outcomes. Furthermore, it is unknown whether chart-based consults (e.g., TACos, e-consults) are as effective as in-person consults. This study suggests, based on surveying hospitalists and residents, that nephrologist recommendations did change management in the majority of cases. Our study points to the need for large-scale, randomized clinical effectiveness trials to assess the impact of TACos on hyponatremia resolution and clinical outcomes.

Future studies should also focus on assessing cost-effectiveness. Billing for inpatient e-consults is now possible by employing interprofessional EHR consultation codes (CPT 99446–99449, 99451), and may offer an opportunity for limited reimbursement for TACos. However, interventions such as the one described are likely to require additional sources of funding, particularly at the time of program launch.^13^ Since our study was a pilot feasibility study, we did not attempt to bill for the nephrologists’ time. We found that the volume of TACos occupied approximately 10% of a single nephrologist’s time, and might require this level of institutional support. These interventions may be cost-effective for health systems if they ultimately prove to result in decreased length of stay or decreased 30-day readmissions.

This study had several strengths, including its randomized design and high survey response rates. It also had limitations, most notably its single-center design and use of a single study nephrologist.

In summary, we found that providing targeted, automatic nephrologist e-consultation for hospitalized patients with hyponatremia was feasible and acceptable to primary teams. Further studies are needed to determine whether this care model leads to improvements in clinical outcomes.

## Supplementary Information

Below is the link to the electronic supplementary material.

Supplementary file1 (DOCX 23 KB)

Supplementary file1 (PDF 136 KB)
