# Supplementary material for: Addressing Inpatient Hyponatremia Through Targeted Automatic E-consults: A Pilot Randomized Trial
Source: J Gen Intern Med. 2024 Oct 22;40(7):1576–83. doi: 10.1007/s11606-024-09054-5 (PMC12052722; doi:10.1007/s11606-024-09054-5)
Supplement: Supplementary file 1 — Supplementary file1 (DOCX 23 KB) [file 11606_2024_9054_MOESM1_ESM.docx]

**Supplemental Table 1: Patient Characteristics in the Intervention and Control Groups, As-Treated Analysis.**

|  | Intervention group  (n = 26) | Control group  (n = 24) | Total  (n = 50) |
| --- | --- | --- | --- |
| Age | 67 [61 – 77] | 61 [57 – 68.5] | 64.5 [60 – 75] |
| Sex | | | |
| Male | 16 (62%) | 11 (46%) | 27 (54%) |
| Race and Ethnicity | | | |
| Non-Hispanic White | 7 (27%) | 11 (46%) | 18 (36%) |
| Non-Hispanic Black | 1 (4%) | 1 (4%) | 2 (4%) |
| Hispanic or Latino | 4 (15%) | 2 (8%) | 6 (12%) |
| Asian | 13 (50%) | 9 (38%) | 22 (44%) |
| Native Hawaiian or Other Pacific Islander | 0 (0%) | 0 (0%) | 0 (0%) |
| American Indian or Alaska Native | 0 (0%) | 0 (0%) | 0 (0%) |
| Other, Unknown, or Declined | 1 (4%) | 1 (4%) | 2 (4%) |
| Primary language | | | |
| English | 16 (62%) | 21 (88%) | 37 (74%) |
| Non-English | 10 (38%) | 3 (13%) | 13 (26%) |
| Insurance | | | |
| Commerical | 1 (4%) | 10 (42%) | 11 (22%) |
| Medicare | 21 (81%) | 7 (29%) | 28 (56%) |
| Medicaid | 4 (15%) | 7 (29%) | 11 (22%) |
| Area Deprivation Index decile* | 1 [1 – 2] | 2 [1 – 3] | 2 [1 – 2] |
| Admission diagnosis category** | | | |
| Certain infections and parasitic diseases | 2 (8%) | 3 (13%) | 5 (10%) |
| Neoplasms | 4 (15%) | 1 (4%) | 5 (10%) |
| Diseases of the blood/blood-forming organs and certain disorders involving the immune mechanism | 1 (4%) | 0 (0%) | 1 (2%) |
| Endocrine, nutritional and metabolic diseases | 4 (15%) | 4 (17%) | 8 (16%) |
| Mental, Behavioral and Neurodevelopmental disorders | 1 (4%) | 0 (0%) | 1 (2%) |
| Diseases of the nervous system | 1 (4%) | 3 (13%) | 4 (8%) |
| Diseases of the eye and adnexa | 1 (4%) | 0 (0%) | 1 (2%) |
| Diseases of the circulatory system | 1 (4%) | 2 (8%) | 3 (6%) |
| Diseases of the respiratory system | 4 (15%) | 2 (8%) | 6 (12%) |
| Diseases of the digestive system | 3 (12%) | 6 (25%) | 9 (18%) |
| Diseases of the musculoskeletal system and connective tissue | 0 (0%) | 3 (13%) | 3 (6%) |
| Diseases of the genitourinary system | 3 (12%) | 0 (0%) | 3 (6%) |
| Symptoms, signs, and abnormal clinical laboratory findings, not elsewhere classified | 1 (4%) | 0 (0%) | 1 (2%) |

Age and Area Deprivation Index decile presented as median [interquartile range]. Percentages may not add to 100% due to rounding.

*State decile. 9 participants had missing Area Deprivation Indices (ADI). Decile 1 is the lowest ADI (least disadvantaged) and 10 is the highest ADI (most disadvantaged).

**Zero participants had admission diagnoses in the following categories: Diseases of the ear and mastoid process; Diseases of the skin and subcutaneous tissue; Pregnancy, childbirth, and puerperium; Certain conditions originating in the perinatal period; Congenital malformations, deformations and chromosomal abnormalities; Injury, poisoning, and certain other consequences of external causes; Factors influencing health status and contact with health services; External causes of morbidity

**Supplemental Table 2: Hyponatremia Resolution and Hospitalization Outcomes, As-Treated Analysis.**

|  | Intervention group  (n = 38) | Control group  (n = 24) | p-value |
| --- | --- | --- | --- |
| **Hyponatremia Resolution** | | | |
| Resolved at discharge ≥ 135 mEq/L (%) | 10 (38%) | 7 (29%) | 0.488 |
| Lowest sodium value  (mEq/L) | 125 [124 – 127] | 126 [124 – 128] | 0.167 |
| Time to hyponatremia resolution ≥ 135 mEq/L (days) | 3.7 [1.8 – 7.5] | 2.9 [2.3 – 4.8] | 0.727 |
| Rate of correction to ≥ 135  (mEq/L/day) | 1.93 [0.96 – 3.65] | 2.24 [0.91 – 3.68] | 0.831 |
| **Hospitalization Outcomes** | | | |
| Length of stay  (days) | 8 [4 – 17] | 6 [3.5 – 14] | 0.442 |
| Number of 30-day readmissions | 0.46 [0.71] | 0.42 [0.58] | 1.00 |
| Cost of hospital stay  ($USD) | $31,492  [15,840 – 69341] | $25222  [13,308 – 62979] | 0.756 |

Continuous variables presented as median [interquartile range] except for readmissions (presented as mean [standard deviation]). Intervention and control groups were compared using chi-squared tests for categorical variables and Wilcoxon rank-sum tests for continuous variables.

**Supplemental Figure 1: Educational framework for hyponatremia diagnosis and management**

*(Please see separately uploaded pdf file)*

**Supplemental Figure 2: Post-intervention satisfaction and knowledge survey instrument.**

1. Were you aware of the nephrologist e-consult for this patient? (e.g. did you get a page, see the note in the chart, or in any other way find out that a nephrologist had reviewed this patient's chart?)

- Yes
- No
- I don't remember

2. Did this nephrology e-consult for hyponatremia change your management? (check all that apply)

- Yes, I ordered diagnostic tests (e.g. urine electrolytes) based on the nephrologist recommendations
- Yes, I ordered therapies (e.g. free water restriction) based on the nephrologist recommendations
- No, the team decided not to act on the recommendations
- No, I had already ordered the recommended tests/therapies when I saw the e-consult

3. How did this nephrology e-consult for hyponatremia affect your understanding of the workup and management of hyponatremia?

- Greatly improved my understanding of these concepts
- Slightly improved my understanding of these concepts
- No effect on my understanding of these concepts

4. Would you like to continue to receive these types of e-consults for your patients with hyponatremia?

- Definitely yes
- Probably yes
- No preference
- Probably not
- Definitely not

1. Any recommendations for how we can improve automatic e-consults for hyponatremia?

________________________________________________________________

1. Which of the following labs should you order to differentiate between an ADH-dependent vs. ADH-independent cause of hyponatremia?
2. Serum sodium
3. Urine sodium
4. Serum osmolality
5. Urine osmolality
6. Urine potassium
7. Which of the following is **true** about **urine sodium**?
8. Urine sodium is low (<20) in volume depletion, heart failure and cirrhosis
9. Urine sodium is low (<20) in syndrome of inappropriate antidiuretic hormone (SIADH)
10. Diuretics may make urine sodium falsely low
11. For **mild and asymptomatic hyponatremia**, which of the following is the most appropriate management?
12. Consult nephrology, urgent correction by 4-6 mEq/L using hypertonic saline, consider DDAVP to prevent overcorrection
13. Give DDAVP, correct slowly (no more than 5 mEq/L per 24 hours) using hypertonic saline
14. Aim for 6 mEq/L correction in 24 hours (maximum 12 mEq/L/24 hours depending on risk of osmotic demyelination syndrome). Method of correction will depend on the cause
15. Aim for 10-15 mEq/L correction in 24 hours. Method of correction will depend on the cause
16. For **severe and symptomatic hyponatremia**, which of the following is the most appropriate management?
17. Consult nephrology, urgent correction by 4-6 mEq/L using hypertonic saline, consider DDAVP to prevent overcorrection
18. Give DDAVP, correct slowly (no more than 5 mEq/L per 24 hours) using hypertonic saline
19. Aim for 6 mEq/L correction in 24 hours (maximum 12 mEq/L/24 hours depending on risk of osmotic demyelination syndrome). Method of correction will depend on the cause
20. Aim for 10-15 mEq/L correction in 24 hours. Method of correction will depend on the cause

*Correct answers to hyponatremia knowledge survey: 6:d, 7:a, 8:c, 9:a*
