# Supplementary material for: Addressing Inpatient Hyponatremia Through Targeted Automatic E-consults: A Pilot Randomized Trial
Source: J Gen Intern Med. 2024 Oct 22;40(7):1576–83. doi: 10.1007/s11606-024-09054-5 (PMC12052722; doi:10.1007/s11606-024-09054-5)
Supplement: Supplementary file 2 — Supplementary file1 (PDF 136 KB) [file 11606_2024_9054_MOESM2_ESM.pdf]

Hyponatremia Workup and Management

Diagnosis

Hyperosmotic S_{Osm}>295
Osmotic substances pull free water into the extracellular space and dilute sodium.
Sugars (Glucose, Sucrose, Mannitol)

Isosmotic S_{Osm} 275 - 295
Laboratory error in measurement.
High Lipids
High Protein (IVIG, Monoclonal Gammopathy)

Hypoosmotic S_{Osm}<275

Urine Osmolality (U_{Osm})
<100 mOsm/kg = ADH Suppressed
Appropriate response in hyponatremia.
>100 mOsm/kg = ADH Active
Either baroreceptor or non-baroreceptor mediated.

Urine Sodium (U_{Na})
<20 mEq/L = RAAS Active
Renal reabsorption of sodium.
>40 mEq/L = RAAS Suppressed or Impaired Response
Renal loss of sodium.

- **Hypovolemia**
  - U_{Osm} >100, U_{Na} <20 → **Non-Renal Volume Loss**: Shock, Hemorrhage, Gastrointestinal Loss
  - U_{Osm} >100, U_{Na} >40 → **Renal Salt Wasting**: Diuretics, Hypoaldosteronism
- **Euvolemia**
  - U_{Osm} <100, U_{Na} <20 → **Excess Water or Low Solute**: Primary Polydipsia, Beer Potomania, Tea and Toast Diet
  - U_{Osm} >100, U_{Na} >40 → **Excess ADH**: SIADH, Hypocortisolism, Hypothyroidism
- **Hypervolemia**
  - U_{Osm} >100, U_{Na} <20 → **Decreased EABV**: CHF, Cirrhosis, Nephrosis
  - U_{Osm} >100, U_{Na} >40 → **Renal Insufficiency**: Severe AKI *can be Hypovolemic*, CKD, ESRD

Easy to fix quickly, watch for overcorrection. (Non-Renal Volume Loss, Renal Salt Wasting, Excess Water or Low Solute)

Takes time to fix, usually slow correction. (Excess ADH, Decreased EABV, Renal Insufficiency)

Framework by John Demko MD, 2020. Document published as part of Hyponatremia TACo Study. Contact timothy.judson@ucsf.edu with questions

Treatment

<u>Goal in all settings</u> is to increase serum sodium by 4-6 mEq/L (<u>maximum 8 mEq/L</u>) per 24-hour period.

Emergency Management

	**Mild-to-Moderate Symptoms**<br>Headache, dizziness, imbalance, confusion, nausea, vomiting, cramps.	**Severe Symptoms**<br>Seizure, coma, pulmonary edema, respiratory arrest, brainstem herniation, death.
**Acute**<br><48 Hours	Any symptoms suggest cerebral edema.<br>**Talk to renal and <u>consider</u> bolus of 3% saline.**	Aim to rapidly increase serum sodium by 4-6 mEq/L over the first 4-6 hours and then maintain constant level for the first 24 hours.<br>**Talk to renal and <u>give</u> 100ml 3% saline bolus.**
**Chronic**<br>≥48 Hours or Unknown	Start general measures.	

Therapy for Correction

1. Treat the underlying process.
   - If you can fix the underlying problem, the sodium will start to improve.
2. Free water restriction.
   - Generally 1.5-2L restriction.
3. Salt tablets if hypo- or euvolemic vs loop diuretics if hypervolemic.
   - Salt tabs started at 1g TID and titrated as tolerated/needed. Can worsen hypervolemia.
   - Loop diuretics are dosed based on severity of volume overload and hemodynamics.
4. Hypertonic saline if severe or refractory.
   - Generally considered if Na <120mEq/L, difficult to correct, and/or any symptoms.
   - Talk to renal!

Increasing HypoNa Severity

Therapy for Overcorrection

1. D5 water bolus and/or infusion.
   - Give water to prevent or reverse overcorrection.
2. Desmopressin injections.
   - Give when patients are at high risk of overcorrection. Talk to renal.
   - Consider with hypertonic saline to avoid unpredictable changes in serum sodium.

Electrolyte Free Water Clearance

$[\mathrm{Na}]_{\mathrm{Serum}} > [\mathrm{Na}+\mathrm{K}]_{\mathrm{Urine}}$

Urine more dilute than serum → lose free water.

Serum sodium will rise.

$[\mathrm{Na}]_{\mathrm{Serum}} < [\mathrm{Na}+\mathrm{K}]_{\mathrm{Urine}}$

Urine more concentrated than serum → retain free water.

Serum sodium will drop.

Expect faster changes in serum sodium if high urine output or large difference between serum and urine electrolytes.
